# Thermogravimetric, Morphological and Infrared Analysis of Blends Involving Thermoplastic Starch and Poly(ethylene-co-methacrylic acid) and Its Ionomer Form

**DOI:** 10.3390/molecules28114519

**Published:** 2023-06-02

**Authors:** Philippe Dony, Françoise Berzin

**Affiliations:** 1 Institue de Thermique, Mécanique et Matériaux (ITheMM), Université de Reims Champagne Ardenne, EA 7548, CEDEX 2, 51687 Reims, France; philippe.dony@univ-reims.fr; 2 Fractionnement des AgroRessources et Environnement (FARE), Université de Reims Champagne Ardenne, INRAE, UMR 0614, 51100 Reims, France

**Keywords:** thermoplastic starch, ionomer, blends, interactions, morphologies, thermal analysis, infrared spectroscopy

## Abstract

This study focuses on the thermal properties and structural features of blends consisting of thermoplastic starch (TPS) and poly(ethylene-co-methacrylic acid) copolymer (EMAA) or its ionomer form (EMAA-54Na). The aim is to investigate how carboxylate functional groups of the ionomer form intervene in blends compatibility at the interface of the two materials and how this impacts their properties. Two series of blends (TPS/EMAA and TPS/EMAA-54Na) were produced with an internal mixer, with TPS compositions between 5 and 90 wt%. Thermogravimetry shows two main weight losses, indicating that TPS and the two copolymers are primarily immiscible. However, a small weight loss existing at intermediate degradation temperature between those of the two pristine components reveals specific interactions at the interface. At a mesoscale level, scanning electron microscopy confirmed thermogravimetry results and showed a two-phase domain morphology, with a phase inversion at around 80 wt% TPS, but also revealed a different surface appearance evolution between the two series. Fourier-transformed infrared spectroscopy analysis also revealed discrepancies in fingerprint between the two series of blends, analysed in terms of additional interactions in TPS/EMAA-54Na coming from the supplementary sodium neutralized carboxylate functions of the ionomer.

## 1. Introduction

Starch is mainly composed of two polysaccharides named amylose (essentially linear) and amylopectin (hyper-branched). Composition is completed by a minor amount of lipids, proteins and ashes [1]. These two polysaccharides are widely used in numerous applications as they are abundant, low cost and renewable and since they can give degradability features to materials if required [2]. Starch can be added to polymeric materials as filler, or plasticized to give thermoplastic starch (TPS) [3]. As for the gelatinization process, plasticizers such as water or glycerol break up the native granule organization [4], separate chains and dismantle double helix conformations, leading to random coil chain state and amorphous material [5,6,7]. At a macromolecular scale, multiple binding situations arise, and chains are swollen depending on the quantity and the nature of plasticizer [8,9]. TPS is sensitive to temperature and humidity variations, inducing with time multiple structural changes, grouped under the term of retrogradation [10,11,12]. This phenomenon implies a crystallinity recovery, taking place on several scale times and affecting dimensional, thermal and mechanical TPS properties [13]. Some additives such as glycerol may attenuate retrogradation, but since they are not chemically bounded, they can exude from TPS or attract more water. To prevent it, several associations with other plasticizers have been tested [14,15]. Lipids and fatty acids interact with amylose by inserting aliphatic segments inside its hydrophobic helix cavity, forming V-amylose conformations [16,17]. Then, parts of complexed amylose chains are hidden from recrystallization behavior, lowering retrogradation. Salts or ionic liquids may also be of interest regarding this phenomenon [10,18]. Nevertheless, only modifying the TPS composition is often insufficient regarding properties of use. Considering packaging applications, some reviews propose solutions to overcome starch and TPS shortcomings [19,20], for example, blending starch, modified or not, with other polymers. As viscosity ratio is a dominant factor in interface handling [21], a first step is to optimize the TPS viscosity in line with the other components by adjusting the plasticizer amount. When viscosity ratio is sufficiently lowered, the size of TPS droplets is lowered and their shape is distorted, giving a thinner and better distributed droplet/matrix morphology, increasing interface. Subsequently, phase inversion is reached at a higher starch percentage and co-continuous morphology may cover a larger percentage of components proportions, depending on the plasticizer amount [22,23]. However, due to thermodynamic and steric considerations, mixing plasticized polysaccharides with hydrophobic polymers is nearly impossible at a molecular level. Then, mixing requires supplementary strategies to increase the components compatibility, essentially by lowering interfacial tension. This can be achieved using chemical modifications of components [24].

A first approach consists of rendering polysaccharides more hydrophobic via chemical modifications to promote compatibility with low polar polymers. Modifications may be obtained through etherification or esterification of alcohol functions [25]. A second approach is to increase the polarity of the second polymer. With polyethylene, a commonly adopted solution is to graft anhydride maleic moieties to obtain PE-g-MA, which is assumed to be located at the interface [26,27]. In such situations, interface saturation determined by emulsification experiments becomes critical [28,29,30]. The underestimated compatibilizer amount does not saturate interface and properties are thus not maximized. If the amount is overestimated, compatibilizer forms micelles and separate from the matrix phase [31]. When used in TPS, glycerol amount is also a factor which must be considered when optimizing the interface [32]. Thus, TPS droplet morphology in blends is directly linked to both percentages of glycerol in TPS and of PE-g-MA compatibilizer [33]. In a controlled situation, the blend can show coalescence inhibition, postponing co-continuous morphology at high TPS amounts, and producing a high elastic network [34,35]. A third approach is to use a polymer already containing hydrophilic moieties, making control of compatibilizer diffusion and saturation useless. However, in many situations, instead of a chemical bonding, a physical network develops. Polyvinyl alcohol is strongly compatible with starch [36]. Ethylene vinyl alcohol copolymer and polyethylene oxide are also good candidates [37]. However, some brittleness develops in blends during aging, corollary to the lack of chemical bindings or strong interactions [38].

Some years ago, a copolymer composed of ethylene and acrylic acid, poly(ethylene-co-acrylic acid) (EAA), was used in TPS/EAA/PE blends to produce blown biodegradable films, with acrylic acid moieties acting as compatibilizer [39]. Subsequent studies highlighted that ethylene segments may interact with polysaccharides like lipids or fatty acid do, binding polysaccharides into a physical network [40,41]. Moreover, in blend with fatty acid, the dimer associations of two carboxylic acid functions from EAA persist and may even be included in the V-amylose complexes [42].

In solution, at elevated pH, the carboxylic acid functions may be transformed into carboxylate ones which may interact with alcohol functions of polysaccharides, stabilize the EAA micelles, decrease coagulation and promote the inclusion complexes [43,44]. Indeed, compatibilization can be helped with ionic molecules due to electrostatic forces acting at molecular level [45].

However, studies were preferably focused on assessing properties of biodegradable TPS/PE blends containing EAA compatibilizer for agricultural mulch and were also focused on EAA inclusion complex formation with polysaccharides conducted in solution. The EAA used was a low mass grafted polyethylene, acting more as a surfactant than a true high mass polymer component such as PVOH or EVOH. Finally, blends have also been made with the help of urea and ammonia, both plasticizing polysaccharides and transforming acid functions into carboxylates in an uncontrolled way.

In the present paper, TPS was blended with a copolymer of poly(ethylene-co-methacrylic acid), named EMAA, and its ionomer derivative, a self-healing polymer where 54% of acid moieties are transformed into carboxylate functions and named EMAA-54Na [46,47]. In such situation, we can take advantages of the following:

1. The capacity of strong interactions coming from inclusion complex formation and ionic plasticization in the case of EMAA-54Na used;
2. The optimization of the viscosity ratio, thanks to glycerol plasticization of starch;
3. Working without a supplementary compatibilizer, which allows us to avoid controlling its dilution and its saturation at blend interface.

Two series of blends were prepared, named TPS/EMAA and TPS/EMAA-54Na. Formulations covered the entire range in percentages allowing us to study phase inversion and the evolution of structural features at interfaces. Differences in results between the two series are interpreted at a molecular level as a supplementary contribution of the carboxylate functions regarding the interface establishment.

## 2. Experimental Procedures

### 2.1. Materials

**Figure 1:**
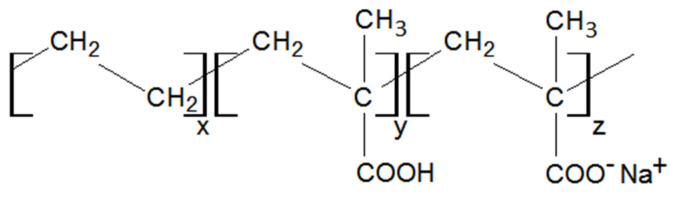
Chemical structure of the neutralized form of EMAA.

Poly(ethylene-co-methacrylic acid) (EMAA) and its ionomer derivative are commercial grades supplied by DuPont company. The composition of these two grades in ethylene (E) and in methacrylic acid (MAA) are very similar. Hence, the two products differ only by the neutralized percentage of the acid function. The percentage of MAA given by the supplier is 9 wt% for EMAA, meaning 3.12% in mole. The ionomer form corresponds to EMAA containing 10 wt% of MAA, meaning 3.08% in mole, and where 54% of COOH functions are neutralized, i.e., the proton is replaced by sodium [48]. Its chemical structure is represented in Figure 1.

When neutralized, EMAA becomes the ionomer EMAA-54Na. The wheat starch was a Natilor grade, supplied by Chamtor company (Bazancourt, France). Glycerol was supplied by Sigma-Aldrich (St. Louis, MO, USA) and both were used as received.

### 2.2. TPS and Blends Preparation

All blends were prepared in an internal mixer (Thermo Scientific Haake Polylab QC, Waltham, MA, USA) equipped with a Rheomix 600 chamber, using roller rotors. The chamber volume is 69 cm^3^.

#### 2.2.1. TPS Preparation

The wheat starch was previously oven-dried at 70 °C for at least 24 h. In an aluminum pan, fresh glycerol and starch were mixed by hand to obtain a homogeneous dough. The maximum duration of this premix process was fixed at 6 min. The dough was then inserted in the mixer at a low temperature (40–50 °C) and low speed (30 rpm) for 2 min. Then, the rotor speed was increased to 100 rpm and the temperature set point was adjusted around 120 °C in order to not exceed 170 °C for the TPS. Starch plasticization was monitored by torque measurement. After reaching a maximum value, it returned to equilibrium, indicating a steady state regime. All mixing times were less than 15 min. After preparation, TPS were stored in desiccators under silica gel.

#### 2.2.2. Blends Preparations

Two series of eight formulations based on TPS/EMAA and TPS/EMAA-54Na blends were prepared through melt mixing with the previously prepared and stored TPS. The maximum temperature of the blends was fixed at 170 °C by adjusting the chamber temperature, with maximum rotor speed fixed at 100 rpm. The chamber was loaded to its maximum capacity and closed by the piston to avoid loss of plasticizer. During insertion of the mass fractions of the two components into the chamber, the rotor speed was fixed at 10 rpm and the set point temperature at 120 °C (above the softening temperature of EMAA). The insertion time was around 2 min. Once the insertion peak had been obtained and the material had melted, the rotor speed was quickly increased to 100 rpm and the set point temperature adjusted (decreased to around 110 °C) to obtain a final temperature of 170 °C for the sample. The maximum mixing time in this condition was less than 10 min. The obtained samples were stored in desiccators under silica gel at room temperature. The targeted compositions in TPS for the two series of blends were 5, 10, 20, 30, 40, 60, 70 and 90 wt%, and blends were labelled from A to G, respectively.

### 2.3. Characterizations

#### 2.3.1. Rheological Behavior of EMMA, Ionomer and TPS

Viscous properties were obtained through the Haake mixer data at 170 °C for the raw components (i.e., the two copolymers and the TPS). The methodology was based on a previous study [49], but adapted in temperature (170 °C) as glycerol was used as plasticizer instead of water. The rotor speed was decreased from 100 rpm to 5 or 10 rpm in five to six steps. For each speed, the temperature was adjusted to maintain the material at 170 °C and the torque was evaluated when the steady state regime was reached. Torque and rotor speed were then transformed into stress and shear rate, respectively [50].

#### 2.3.2. Scanning Electron Microscopy (MEB)

Low magnification scanning electron microscopy (×500) was performed using an ITACHI TM-1000 microscope. Prior to the observations, samples were broken in liquid nitrogen and the surface was observed with no further modification.

#### 2.3.3. Thermogravimetric Analysis

Mass losses were measured with a TGA Netzsch TG 209 F3 piloted by Proteus 6 version. Sample weights varied from 9 to 15 mg. The heating rate was fixed at 5 °C/min from ambient to 550 °C under nitrogen atmosphere. Thermograms (TG) and first derivative (dTG) data were exported in Excel format for analysis. The methodology of mass loss analysis used a fitting procedure [51], where TG traces were approximated by a sum of sigmoid curves, according to Equation (1):

(1) $$∆m=m_{u}-m_{l}*\left[\frac{1}{\left(1+exp\left(\frac{T-\theta_{1}}{\theta_{2}}\right)\right)}\right]$$

where *m_u_* and *m_l_* are, respectively, the upper and lower limits of mass percentage of the applied sigmoid. *T* is the temperature and *θ*_1_ and *θ*_2_ are parameters analogous to temperature. In an ideal situation, where mass losses are well separated, *θ*_1_ is related to the temperature corresponding to the peak of the dTG curve. Its value corresponds to the inflexion point of the sigmoid. As real conditions do not give separated mass losses, *θ*_1_ obtained by approximation did not correspond exactly to the dTG peak. The temperature *θ*_2_ is related to the spreading of the sigmoid. If the curve shows significant temperature spreading, the value of *θ*_2_ is high. Optimization was performed with the Levenberg-Marquardt type algorithm supplied by the Excel worksheet. For each sigmoid curve, the estimated parameters of Equation (1) were provided to the algorithm. Then, a TG curve was fitted by applying the convergence algorithm, based on the minimization of the least square between the sums of all sigmoids and the TG curve. A correlation coefficient similar to that used in a non-linear regression was calculated in order to check the fitting performance.

#### 2.3.4. Fourier Transform Infrared Spectroscopy (FTIR)

A Shimadzu IR Affinity-1S equipped with a Miracle™ single attenuated total reflection (ATR) cell from Pike Technologies was used to sample the absorbance of TPS, EMAA, ionomer and blends. A diamond/ZnSe cell was mounted on the ATR. For each spectrum, 32 or 64 scans were performed at a resolution of 4 cm^−1^ from 650 cm^−1^ to 4000 cm^−1^. Multiple point baseline correction was applied to all samples to minimize baseline drift. Several spectral band resolutions were obtained thanks to Fityk 1.3.1, a peak fitting software. The shapes of individual bands were modelled by Gaussian distributions, the sum of bands being used for calculation of the nonlinear fit. The convergence of the calculation was based on minimizing the weighted sum of squared residuals and was ensured by the iterative Levenberg-Marquardt algorithm. The relevance of the calculation was framed by the weighted sum of squared residuals value (WSSR). All fits showed a WSSR below 10^−3^.

## 3. Results and Discussion

### 3.1. Viscosity Ratio Evaluation

**Figure 2:**
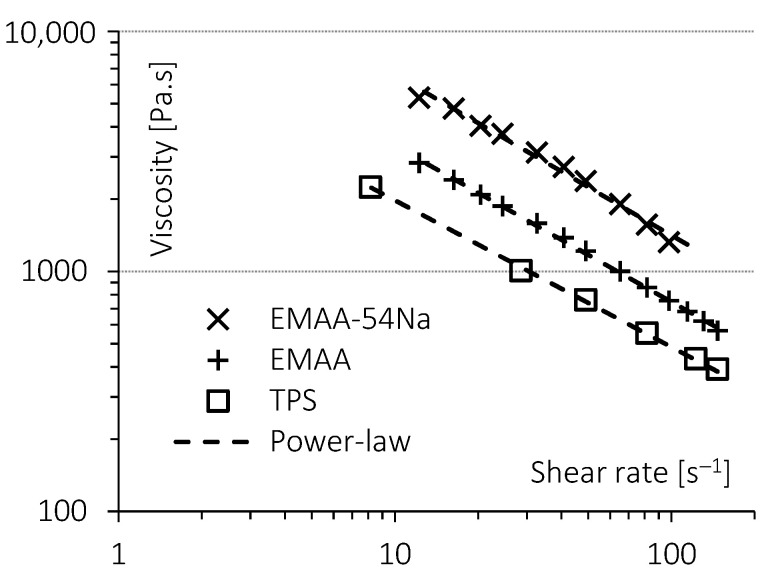
Viscosity curves at 170 °C of TPS, EMAA and EMAA-54Na.

The viscosity ratio of the raw components is one of the factors affecting the blends morphology. When the viscosity ratio is close to unity, a fine dispersion of the minor component in the matrix may be obtained [23,52]. Viscosity measurements of the raw components are shown in Figure 2. At 100 rpm, the estimated shear rate in the internal mixer is 82 s^−1^. Each viscosity curve can be correctly fitted by a power-law (R^2^ values were above 0.988 for the three fits). TPS showed the lowest viscosity in this shear rate range. The viscosity ratio is given by Equation (2):

(2) $$x=\frac{\eta_{d}}{\eta_{m}}$$

where *η_d_* and *η_m_* are the viscosities of the dispersed phase and of the matrix, respectively. For non-Newtonian fluids, this ratio depends on the shear rate. In the present case, the viscosity curves of the three components are parallel, and thus this ratio is quite constant.

Values obtained at 82 s^−1^ are given in Table 1. When TPS was used as the matrix, the value of the ratio was greater than one. On the contrary, when TPS was the dispersed phase, the value of the ratio was less than one. However, these values remained close to unity, indicating an appropriate choice for blend components.

### 3.2. Thermal Properties

TGA provided the degradation temperature range of all basic components and of all blend formulations, TPS/EMAA and TPS/EMAA-54Na. Based on the TGA thermograms of basic components and using a simple additive law, theoretical thermograms can be built for all formulations, which are representative of blends composed with fully incompatible materials [53]. The curves of real blends were fitted with the methodology detailed in Section 2.3.3.

#### 3.2.1. Blend Components

**Figure 3:**
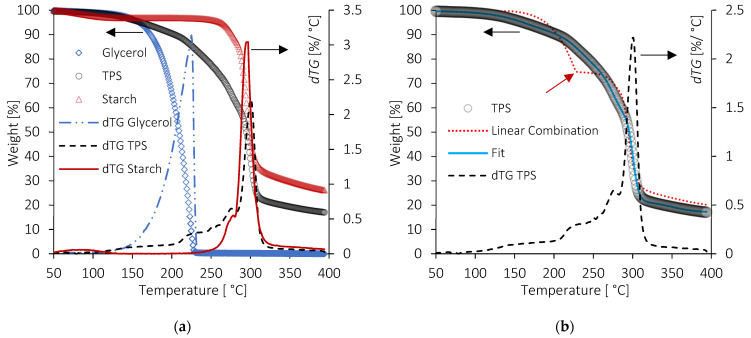
(**a**) TG and dTG curves of glycerol, starch and TPS and (**b**) TG and dTG of TPS, the TG fit of the TPS, and linear combination of starch/glycerol (74.8/25.2 in wt%). The red arrow indicates the end of glycerol evaporation and the beginning of starch degradation in the thermogram obtained by linear combination.

Figure 3a shows that glycerol evaporated first with an onset at 150 °C; the process was completed before 250 °C with no residue. Wheat starch showed three mass losses. The first one, due to the remaining free water, was visible around 80 °C. With our drying procedure condition, it represents 3.2% of the total mass. The other two mass losses were associated with polysaccharides. The onset degradation temperature of starch was visible around 250 °C. The main decomposition followed with a small loss under 300 °C and a maximum rate observed at 300 °C. A residue around 26% remained at 400 °C. These results are in accordance with those observed by several authors [54,55]. The decomposition of TPS and its fit are shown in Figure 3b and agree with previous observations [56]. The frontier between mass loss of glycerol and starch detected by the fitting is indicated by a red arrow on Figure 3b and gives the weight composition in starch/glycerol of the TPS: 74.8/25.2 (wt%).

When the same weight ratio was used in a linear combination, the curve, corrected for the presence of the remaining water coming from the starch, led to a plateau between glycerol and starch decompositions. This plateau cut the experimental curve just above the beginning of the starch degradation found by the fit.

The shape of the curve estimated with a linear combination differed from the TPS one. Because of the intimate mixing of TPS components at the molecular level, due to strong interactions between starch and glycerol, mass losses are not well separated. This leads to a decomposition temperature of the glycerol above its usual endset temperature and a more gradual mass loss. To a lesser extent, the same analysis could be performed for starch but in the opposite direction. Its decomposition may occur at a lower temperature than expected, making it more difficult to estimate the frontier between the starch and glycerol range of weight loss. This drawback, i.e., a situation where components are not clearly separated due to their close decomposition temperatures, was overcome with the help of the method described in Section 2.3.3.

EMAA and EMAA-54Na were also subjected to fitting. Products were stable until 400 °C. Only volatiles were lost below this temperature, representing 2.8% of mass loss for EMAA and 3.8% for EMAA-54Na. The ionomer was able to absorb water through carboxylate functions that could participate in the supplementary mass loss. The two copolymers showed multistage decompositions between 400 °C and 490 °C [57]. Above 490 °C, EMAA showed a residue of around 1%. Due to the presence of sodium carboxylate functions, the residue of the ionomer was greater and reached 6%. It is believed that it was composed of sodium carbonate [58]. The last maximum rate of decomposition was observed at 456 °C and 449 °C for the ionomer and EMAA, respectively.

#### 3.2.2. TPS/EMAA and TPS/EMAA-54Na Blends

In totally immiscible or weakly miscible blends with well-separated degradation temperatures, composition is easily estimated by TGA. Experimental TG curves and linear combinations (LC) overlap well, interfaces between components are rough, limited and the features of raw components are kept. With miscible or compatible materials such as starch and glycerol in TPS, however, new strong interactions arise at the interface. The minor phase, if it remains, is finely dispersed, and the TG curves of blends deviate from those made from the linear combinations of raw components [59]. In that situation, the temperature range of the mass loss may be shifted by varying the composition and nature of raw components [60]. In a situation of total miscibility, decomposition temperatures merge [61]. The shifts of the main decomposition temperatures of components in blends compared to the original ones depend on the synergy between them. During thermal decomposition, chemical reactions occur, leading to the stabilization or destabilization of products. Each mixing situation is unique, and caution must therefore be exercised in analyzing data [62].

**Figure 4:**
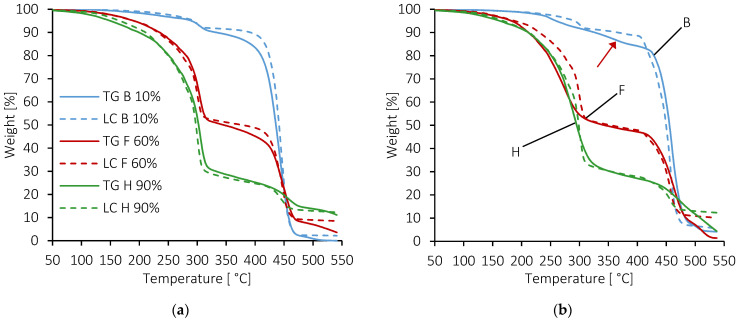
TG and linear combination (noted LC) of formulations B (10 wt% TPS), F (60 wt% TPS) and H (90 wt% TPS) for (**a**) TPS/EMAA and (**b**) TPS/EMAA-54Na. The red arrow points to the occurrence of the intermediate mass loss between the two main losses associated with TPS and ionomer.

**Figure 5:**
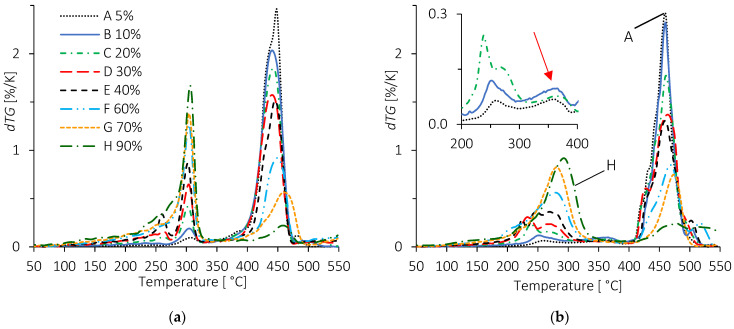
dTG curves of all formulations for (**a**) TPS/EMAA and (**b**) TPS/EMAA-54Na. Percentages are relative to TPS in weight. The red arrow has the same meaning as in Figure 4b.

It can be seen on Figure 4 and Figure 5 that the onset temperatures of degradation of the two copolymers, around 400 °C, are far above the endset temperature of TPS. The degradation temperature ranges of the components do not overlap, confirming that the phases remain separated and indicating a slightly compatible situation. Figure 4 and Figure 5 also show some discrepancies in TPS degradation behavior between the two blends. Concerning the compatibility and influences of components on each other, the key for interpreting Figure 4 and Figure 5 is the position of the LC curves compared to the experimental TG ones. When the latter are under the LC ones, it indicates a destabilization effect on the degradation behavior of the component in the blend.

A first remark that can be made about Figure 4 is that above 300 °C, the char formed by the polysaccharide continues to lose weight in the range of temperature associated with the decomposition of EMAA or ionomer. For the two blends, the final residue at 550 °C may be composed of the two components and thus divided into two parts, as well. A second remark concerns the shape of the TG curves. Deviations from LC were observed in all formulations, indicating interactions between TPS and copolymers. Moreover, the two series of blends do not look alike, meaning that the interactions may be different.

For the TPS/EMAA blends (Figure 4a), the TPS decomposed at 300 °C with little to no deviation of its temperature. Differences between experiments and LC were small. On the contrary, for TPS/EMAA-54Na, a clear deviation was observed for the TPS decomposition temperature in the B and F formulations under 300 °C (Figure 4b). Experimental curves were lower than the LC ones, meaning that interactions seem to destabilize the TPS phase by lowering its thermal properties. A close look at formulation B of the TPS/EMAA-54Na blend (Figure 4b) shows a small inflexion of the TG curve around 350 °C (red arrow), between the decomposition temperatures of starch and EMAA-54Na, indicating the decomposition of a possible intermediate structure.

This inflexion is not clearly seen in the B formulation of the TPS/EMAA blend (Figure 4a), even though a strong deviation exists at the onset of the main degradation temperature of EMAA in B and F blends, around 400 °C. In the H formulation of the two series of blends, TPS is the main component and only small to no deviation seems to occur before 450 °C, as expected. On the contrary, in the temperature range of copolymer degradation, the slopes of the H experimental curves are less pronounced than in the linear combination. This may be explained by a phase inversion and the encapsulation of EMAA or EMAA-54Na by the char formed by the TPS, delaying and slowing its decomposition [63]. For the B and F formulations of TPS/EMAA-54Na, deviations in the temperature range of TPS decomposition lead to a strong destabilization of TPS minor phase in comparison with LC curves, which will be better observed through the following dTG curve study (Figure 5).

The dTG curves of Figure 5a confirm that the analyses from Figure 4a and TPS/EMAA blends show sharp peaks around 300 °C, similar to TPS alone. In addition, as the TPS percentage increases, small shoulders and peaks develop under 300 °C, indicating creation of a glycerol-rich polysaccharide phase. This behavior comes to reinforce an already proposed model [32]. This situation expands for TPS/EMAA-54Na blends (Figure 5b), where the TPS degradation temperature range is divided into multiple peaks that evolve with the percentage of TPS. At a low TPS content (formulation A), a first decomposition is seen near 250 °C and precedes a second one above 350 °C (insert in Figure 5b). This inflexion also appears in the B and C formulations.

When the percentage of TPS increases, the low temperature decomposition peak around 250 °C splits and the higher temperature shoulder expands. Its maximum temperature increases from 250 °C in the A formulation to reach 300 °C for the H one, while the peak at 350 °C is reduced and eventually disappears. This suggests that a small part of the TPS is destabilized, i.e., more plasticized, and starts its decomposition at a lower temperature (250 °C) than expected. We may suggest that EMAA-54Na replaces the glycerol in its plasticizing role of polysaccharide chains. The liberated glycerol may overplastify polysaccharides chains, constituting the enriched glycerol TPS domains. A second part of TPS is strongly associated to the ionomer and decomposes at an intermediate temperature (350 °C). Obviously, when the quantity of TPS increases (and thus the amount of ionomer decreases), the proportion of TPS bonded to ionomer decreases proportionally, and the deviations around 250 °C and 350 °C tend to disappear, while the TPS degradation temperature returns to its expected value of 300 °C. It is to be noted that in the TPS/EMAA-54Na blends, peaks associated to TPS degradation occur over a wider temperatures range than in the TPS/EMAA one, reinforcing the hypothesis of stronger interactions with EMAA-54Na. The maximum peak temperatures of EMAA and EMAA-54Na increase slightly due to the screening of the TPS char, as the latter increases in proportion.

**Figure 6:**
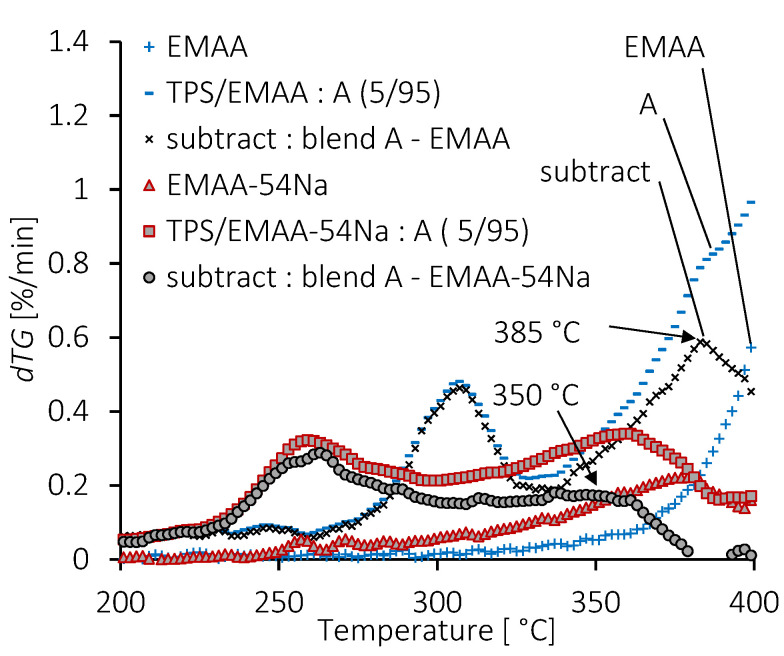
dTG curves of EMAA, EMAA-54Na, their blends with 5 wt% of TPS, and subtracted curves between the copolymer EMAA or EMAA-54Na and its relative blends.

The weight loss occurring between 300 °C and 400 °C visible in the A, B and C formulations of TPS/EMAA-54Na blends (arrow in Figure 5b) may also exist in TPS/EMAA. To check this, the EMAA experimental dTG curve was subtracted from that of the A blends (5 wt% of TPS). The results, also for TPS/EMAA-54Na, are shown in Figure 6. The subtraction eliminates the weight loss of EMAA or EMAA-54Na in the corresponding A blends, revealing the intermediate degradation temperature peak. The temperature associated with this degradation in TPS/EMAA-54Na is lower (350 °C) than that in TPS/EMAA (385 °C), confirming the destabilizing thermal effect of the carboxylate functions on TPS decomposition. Associations between starch and poly(ethylene-co-acrylic acid) (EAA) were detected through different techniques and were due to a particular complexion interaction of EAA polyethylene main chain segments inside the polysaccharide helix [43]. This kind of interaction resembles those encountered between fatty acids and polysaccharides.

TPS engaged in such a complexion interaction may resist temperature degradation differently in TPS/EMAA-54Na blends compared to TPS/EMAA. As the only structural difference between the two blends is the neutralization of 54% of the carboxylic acid in EMAA-54Na, it can be assumed that carboxylate functions are responsible for the differences observed between the two blend series. Based on this assumption of a strong interactions existing at the interface between polysaccharides and copolymers, Figure 6 indicates that the interactions are of different nature, where the carboxylate group in EMAA-54Na is responsible for the weakening of the interface thermal property, seen at 385 °C in the A blend of TPS/EMAA, but at 350 °C in TPS/EMAA-54Na.

### 3.3. Morphology of Blends

**Figure 7:**
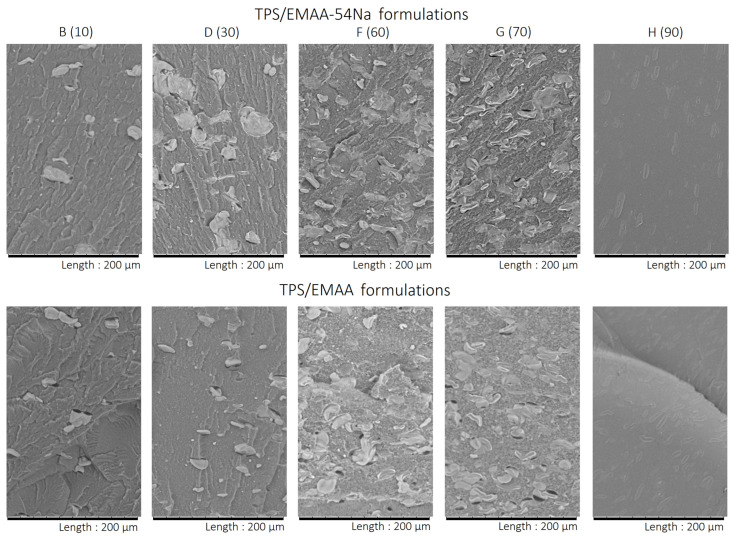
SEM images for formulations B, D, F, G and H of TPS/EMAA-54Na (**top**) and TPS/EMAA (**bottom**). wt% of TPS in brakets.

In the SEM images, contrast variations in grey at low magnification for blends B indicate that TPS appears in a clearer domain than the copolymer EMAA or its ionomer (Figure 7). Light grey TPS inclusions are visible in the copolymer matrices for formulations B to G and are detailed as ghosts, an insoluble part of TPS [64,65]. When the percentage of TPS increases, the inclusions are more numerous. We observe also a distinct evolution in the roughness of the fractured copolymer surface, becoming less and less pronounced in TPS/EMAA with the increase in TPS amount, compared to TPS/EMAA-54Na. Alongside the inclusions phase, a second TPS distribution size may exist, more finely dispersed in matrices, invisible at this magnification and coarser in TPS/EMAA (Sample F and G in Figure 7). Unlike the morphologies of blends B to G, the two H formulations show a totally different pattern. This reveals that the phase inversion point is situated between formulations G and H, i.e., between 70 and 90 wt% of TPS [34]. At 90 wt%, the TPS becomes the matrix and encapsulates the EMAA or the ionomer, explaining the increase in degradation temperatures for the H formulations seen in Figure 4 and Figure 5. For these H formulations, at this magnification, the copolymer or the ionomer are invisible. Note however that, in H formulations, rounded starch inclusions previously dispersed in copolymer matrix are still visible and are embedded in an uniform TPS matrix, meaning that before the phase inversion, truly gelatinized polysaccharide was thinly diluted in copolymers thanks to the compatibilization capability of the two copolymers [29].

### 3.4. Structural Features of Blends by FTIR Analysis

**Figure 8:**
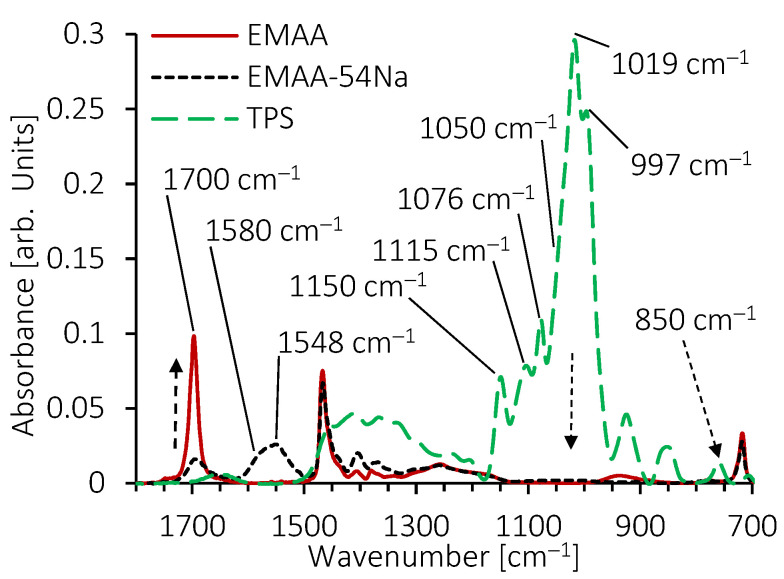
FTIR spectra of EMAA, EMAA-54Na and TPS, from 650 cm^−1^ to 1800 cm^−1^.

FTIR spectroscopy is a useful methodology to investigate chemical structures and interactions at short length scales. The main vibration modes of the TPS, EMAA and EMAA-54Na spectra under 1800 cm^−1^ are identified in Figure 8. The superimposition reveals three areas of bands where products show nearly no absorbance overlay between each other, allowing the impact of one product on the other to be interpreted without any interferences.

The first range concerns bands between 1600 cm^−1^ and 1800 cm^−1^, assigned to (C=O) stretching vibration of the acid form of the carboxyl group existing in EMAA and EMAA-54Na. In this range, the only band which may interfere with the analysis is the one at 1650 cm^−1^ assigned to (O-H) bending vibration of the alcohol group of polysaccharides, glycerol and to the bonded water. The second range is between 1500 cm^−1^ and 1600 cm^−1^ and includes asymmetrical vibration modes of the carboxyl group (COO^−^) in EMAA-54Na only [66]. Bands are not resolved, and a maximum appears around 1548 cm^−1^. The third range corresponds to the 950–1180 cm^−1^ fingerprint associated with polysaccharide bands. It is composed of highly coupled vibration modes of (C-O), (C-O-C) and (C-C) stretching and (C-OH) bending. Again, in this third wavenumber area, EMAA and EMAA-54Na show almost no absorption. As a result, in blends, by focusing on the evolution of bands in the first two domains between 1500 cm^−1^ and 1800 cm^−1^, we can observe the consequences of TPS on the vibration modes of the (C=O) acid group in EMAA and EMAA-54Na and on the (COO^−^) carboxyl group in EMAA-54Na. Similarly, we can observe the impact of EMAA or EMAA-54Na on the polysaccharide vibration bands between 950 cm^−1^ and 1180 cm^−1^.

The superimposition of the FTIR spectra of the blends provides three indications. Firstly, the maxima of the (C=O) stretching vibration observed at 1700 cm^−1^, which is assigned to a dimer formation composed of two carboxylic acid functions associated by hydrogen bonding, remains in its position whatever the amount of TPS. This indicates that carboxylic acid functions remain under this form in both blends, TPS/EMAA and TPS/EMAA-54Na. This hypothesis must be moderated, however, because their absorbance is low in TPS/EMAA-54Na blends and because some of these acid functions may be involved in the formation of multiplets [67]. Secondly, the overall poorly resolved shape of the TPS fingerprint, between 950 cm^−1^ and 1180 cm^−1^, changes with the amount of TPS. When the latter decreases, a shoulder of the 1019 cm^−1^ band, which had almost disappeared, can be seen emerging around 1050 cm^−1^, indicating an impact of EMAA and EMAA-54Na on the configuration of the polysaccharide chains. The shape of the 1115 cm^−1^ band is also modified. Thirdly, with increasing quantities of TPS, the shoulder visible at 1580 cm^−1^ seems to disappear more quickly than the maxima located around 1548 cm^−1^. A local disruption of the environment around the carboxyl function in TPS/EMAA-54Na can be a consequence of its interaction with TPS and may cause the disturbance in the asymmetric stretching (C=O) vibration of the (COO^−^) group.

**Figure 9:**
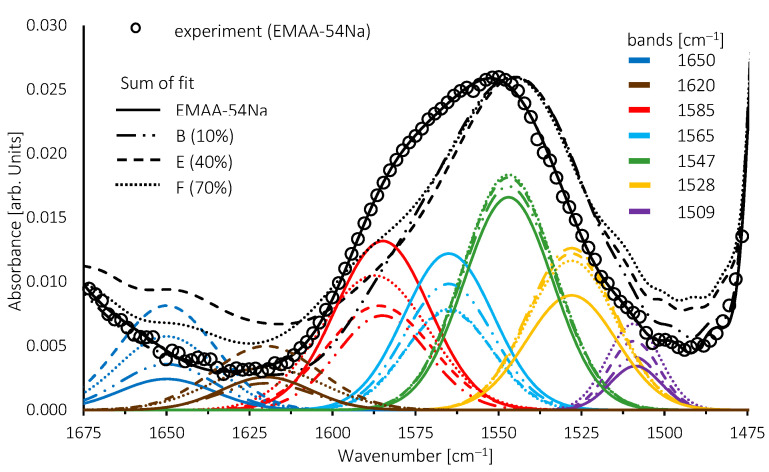
Band fitting of EMAA-54Na and TPS/EMAA-54Na with 10% (B), 40% (E) and 60% (F) of TPS in weight. Curve with rounded dots is for the experimental EMAA-54Na spectrum only.

To investigate the last point, an overlay of some spectra of TPS/EMAA-54Na and their sets of bands obtained by fitting are shown in Figure 9. Prior to the fitting experiment, the interference of the TPS product was minimized by subtracting TPS spectra from the blend one. Subtraction was monitored by the disappearance in the blends of the band at 850 cm^−1^, solely due to TPS (Figure 8). The fit was also performed below 1500 cm^−1^ and above 1600 cm^−1^ in order to consider the contributions of adjacent bands. The band positioning is based on the literature and is part of the following discussion. In order to compare the band contributions to the sum, as well as to take into account the overall decrease in the bands with the decreasing proportion of EMAA-54Na in the blends, all the spectra were normalized on the 1547 cm^−1^ band, the major contribution to the sum of fit.

The spectra of EMAA-54Na, blends B, E and F, were fitted with five bands. Additionally, the evolutions of two other bands at 1650 cm^−1^ and 1620 cm^−1^ are shown. The first one is assigned to the vibration mode of (O-H) from water in EMAA-54Na. The second band is assigned to the (C=O) vibration mode of acid functions associated with the carboxyl group. They form acid salt compounds [68].

Two bands fixed at 1547 cm^−1^ and 1568 cm^−1^ correspond to sodium surrounded by a 6-coordination carboxyl configuration [69]. In our case, a better fit is achieved with a band at 1565 cm^−1^. The band existing at 1585 cm^−1^ is assigned to a 4-coordination (COO^−^) configuration around the cation [66]. This band is dominant in Zn^2+^ neutralized ionomers because of its strong and well-designed complexion with (COO^−^) groups. As a result, zinc-based ionomers have been widely used for the study of 6- and 4-coordination complex configurations through FTIR [68]. This 4-coordination complex configuration may exist in Na ionomer, and the band is added to the fit [67,69,70]. A fourth band at 1509 cm^−1^ was added where the EMAA-54Na spectrum shows a shoulder. The frequency of the 1547 cm^−1^ band diminishes to 1540 cm^−1^ when water is added [71]. Then, to take into account this evolution and the shoulder appearing in blends under 1540 cm^−1^, a fifth band centered at 1528 cm^−1^ was added to perfect the fit. A close look at other alkali metal cation (Cs+, Li+) ionomers shows that vibration modes exist under 1540 cm^−1^, even if they were not exploited [69]. The frequencies used for these fifth bands are in agreement with those found in a recent study [70].

Water, temperature, pressure and neutralization percentage have been shown to change the fingerprint spectrum in this region [67,71,72,73]. Simultaneous neutralization by more than one cation also modifies the spectrum [66,74,75]. It was concluded that this is due to local disturbance of the configurations of the coordination complexes. Some vibration modes are enhanced while others are reduced, modifying the overall shape of this region of the spectrum.

When the percentage of TPS increases, the two bands with the strongest frequencies at 1585 cm^−1^ and 1565 cm^−1^ decrease while the three lowest ones at 1547 cm^−1^, 1528 cm^−1^ and 1509 cm^−1^ increase slightly. Above 40 wt% of TPS, the opposite behavior is observed for the 1585 cm^−1^ band, which starts to rise again. When the percentage of TPS increases, the 1625 cm^−1^ and 1650 cm^−1^ bands also increase. All these results indicate a disturbance of the sodium coordination complexes configurations caused by new interactions with TPS. The decrease in absorbance of the higher wavenumber bands signs the weakening of the carboxylate group bonds, indicating H-bonding with alcohol functions of polysaccharides or glycerol. However, at this point, it remains hasty to propose specific chemical interaction configurations.

**Figure 10:**
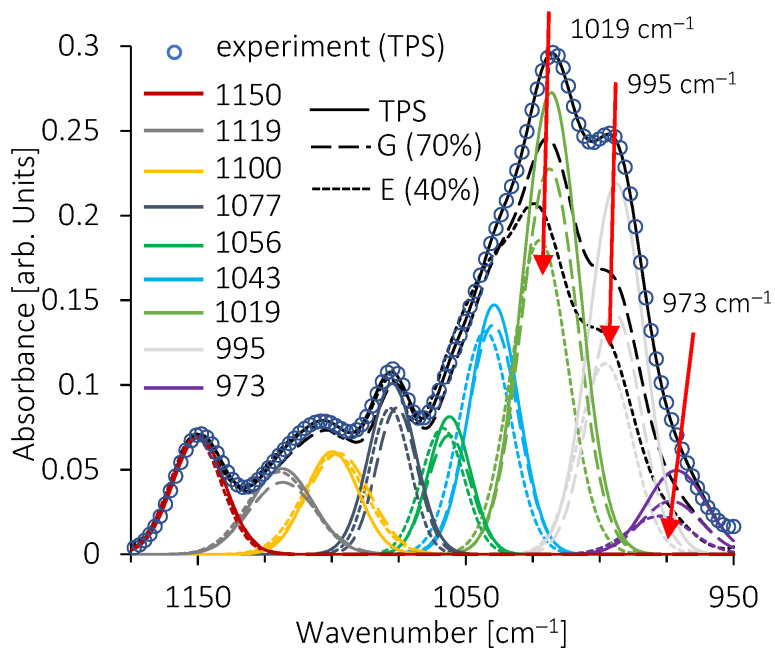
Bands fitting of the polysaccharides fingerprint, curves of G (70 wt% TPS) and E (40 wt% TPS) blends of the TPS/EMAA series. Curve with rounded dots is for the experimental TPS spectrum only.

**Figure 11:**
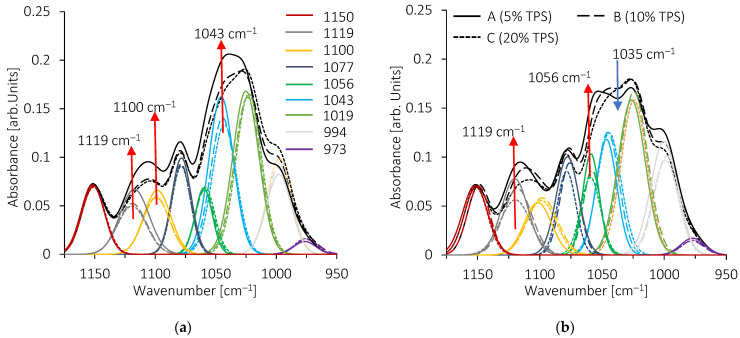
Bands fitting of the polysaccharide fingerprints, curves of A (5 wt% TPS), B (100 wt% TPS) and C (20 wt% TPS) blends of (**a**) TPS/EMAA series and (**b**) TPS/EMAA-54Na series.

In the polysaccharides fingerprint, curves fitting shows multiple bands from 952 cm^−1^ to 1178 cm^−1^ (Figure 10 and Figure 11). Frequency values are in good agreement with those previously reported by some authors [76,77]. To account for the decrease in absorbance with the decrease in TPS amount in the blends, all the spectra were normalized to the vibration band at 1150 cm^−1^. The spectrum of pure TPS shows bands associated with a gelatinized state [78,79], with a well-developed band at 1019 cm^−1^ and the shoulder at 995 cm^−1^. For both blends, TPS/EMAA and TPS/EMAA-54Na, the absorbance of these two bands decreases strongly with a decrease in the TPS amount (Figure 10).

**Figure 12:**
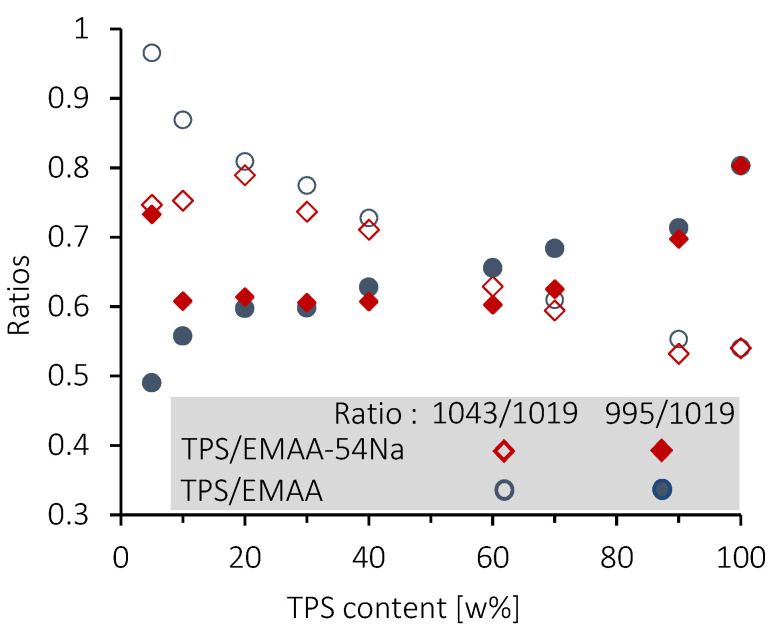
Evolution of infrared absorbances ratios 1043/1019 and 995/1019 as a function of TPS content (wt%) for the two series of blends.

When the amount of TPS is below 20 wt% (C formulation), the 1119 cm^−1^ band increases in the two series of blends. For the TPS/EMAA-54Na blend, an increase in the absorbance of the 1056 cm^−1^ band is observed, while for TPS/EMAA, the increase is observed for the 1043 cm^−1^ and 1100 cm^−1^ bands (Figure 11). Discrepancies in fingerprint evolution reveal differences in interactions from the polysaccharide point of view between the two blends, caused by the carboxyl (COO^-^) group in EMAA-54Na, which does not exist in EMAA. In the TPS fingerprint, it is impossible to assign an individual band to a specific vibration mode of chemical bonds, but the band ratios were used to evaluate and qualify the polysaccharides configuration states (i.e., amorphous or ordered) [80]. Evolutions of these ratios are shown in Figure 12. The 1047/1022 absorbance ratio (1043 cm^−1^ to 1019 cm^−1^ in our case) was used to quantify the crystalline state [77]. FITR is unable to determine the type of long-range order, i.e., to distinguish between the B or A type of helix packing [80]. However, it determines the relative quantity of short-range order against an amorphous configuration, and thus indirectly the crystalline amount.

Regarding the 1047/1022 absorbance ratio (R_1_), and starting from 100% of TPS, as the percentage of EMAA or EMAA-54Na increases, the frequencies associated with a disorganized state of polysaccharide chains decrease and those related to a retrograded or crystallized state increase. The configurations of the chains evolve from a disorganized state (R_1_ ≈ 0.55 at 100 wt% of TPS) to a more ordered one with the presence of EMAA or EMAA-54Na (R_1_ ≈ 0.8 at 20 wt% TPS for both series of blends). This evolution can be correlated to the capacity of polysaccharide chains to complex ethylene segments of EMAA or EMAA-54Na inside hydrophobic cavity of helix conformations at interface. Indeed, the less the TPS amount, the more the remaining one is short-range ordered. This evolution means that the polysaccharides located at the interface, becoming proportionally more important with the decrease in TPS amount, are more short-range ordered due to their contacts with copolymers. Dimer acid could also be included in the helix cavity as already suggested [42]. Participation of dimer acid groups to inclusion complexes could explain why the local structural configuration of the polysaccharide chains is moved to a more ordered state, while acid dimer groups are not structurally changed. There is a split at a low TPS level under 20 wt%. We observed a reversal behavior with a decrease in the R_1_ ratio (R_1_ ≈ 0.75 at 5 wt% of TPS) for the TPS/EMAA-54Na blends. This implies an upturn to a less organized polysaccharide chain configuration. It reveals the role of the carboxyl COO^-^ groups on TPS, which prevents an increase in crystalline quantities via complex inclusion. Carboxylate functions act as physical reticulation points or, if not engaged in multiplets, as head of fatty acid, preventing a pronounced insertion of ethylene segments of EMAA-54Na into the hydrophobic helix cavity of polysaccharides as the acid dimers do. In comparison, if carboxylate functions do not exist in EMAA, then polysaccharide chains can complexify ethylene segments easily, increasing the short-range order. Then, the ratio continues to increase in TPS/EMAA series, until it reaches R_1_ ≈ 1.

The R_2_ absorbance ratio 1000 cm^−1^ to 1022 cm^−1^ (995 cm^−1^ to 1019 cm^−1^ in our situation) reveals the quality of helices packing (i.e., alignment of helices, in short-length scale) according to different plasticization and moisture situations [79]. Beginning with 100 wt% TPS, this ratio shows a very low value (R ≈ 0.83) in good agreement with a gelatinized starch containing a low number of plasticizers. In such situations, packing helices are already highly disorganized. A diminishing ratio is associated with a more distorted packing, while a higher ratio value corresponds to a better packing state of short helices. We observed the expected decrease in the ratio with the increased quantity of hydrophobic EMAA and EMAA54-54Na. Copolymers disrupt the helix packing at the interface, but, as for the R_1_ ratio, a divergence in behavior is observed between the two blends. The ratio increases again in TPS/EMAA-54Na under 10 wt% of TPS, meaning that the carboxylate group intervenes at low TPS amount by organizing the stacking of the low quantity of polysaccharide chains and helices at the interface.

## 4. Conclusions

In this paper, we studied the properties of blends of thermoplastic starch (TPS) with poly(ethylene-co-methacrylic acid) copolymer (EMAA) and with its ionomer form (EMAA-54Na). Structurally, these two grades of copolymer differ only by the percentage of carboxylic acid functions neutralized by sodium in the ionomer (54 wt%). Sigmoid functions were used to fit TG curves in order to confirm the composition of formulations of the two blends. By assuming that complex interactions exist in both TPS/EMAA-54Na and in TPS/EMAA blends, the differences in temperature properties detected by TGA can be explained by the participation of the carboxylate functions in the interactions. The thermal resistance of a portion of the TPS involved in the inclusion may be altered either by the direct participation of the carboxylate functions in the inclusion or by their indirect participation through the presence of physical crosslinking (multiplets, clusters), or by new interactions between polysaccharide and carboxylate. In that way, a portion of TPS may degrade in two different temperature ranges. We hypothesize that in the two blends and for low percentages of TPS (formulations A, B and C), the mass loss observed at 350–400 °C may correspond to part of the remaining interface structure. SEM images confirmed the phase inversion around 80 wt% of TPS. Dissimilarity in surface roughness indicates a possible second smaller size distribution of TPS particles dispersed in the matrix and a difference in the fineness of these TPS particles. Finally, the infrared experiments showed the existence of interactions between the two components. At a low percentage of TPS (under 20 wt%), differences in spectra and ratios evolution between the two series reveal involvement of carboxylate function from EMAA-54Na into the interface construction. Interaction patterns are distinct due to carboxylate groups. COO^−^ functions maintain a low short-range order quantity (R_1_ ≈ 0.75), but promote a better packing of helices (R_2_ ≈ 0.75), indicating that the complexion of ethylene segments into hydrophobic helix cavities of polysaccharides may be limited. At the opposite, thanks to insertion of ethylene segments of EMAA into hydrophobic cavities of polysaccharide helices, the quantity of short-range order is high (R_1_ ≈ 1), but packing perfection is lower (R_2_ ≈ 0.5). At high percentages of TPS, polysaccharides involved in the interface are under-represented compared to ones located inside droplets and are hidden from copolymers. Then, the ratio evolutions of the two series of blends become similar, giving a parallel decrease in short-range order amount and helices packing quality with EMAA or EMAA-54Na. As in the TGA and SEM experiments, the infrared imaging shows differences in results between the two sets of formulations that can be explained by the presence of carboxylate functions.

## Figures and Tables

**Table 1:** Viscosity ratios of TPS, EMAA and EMAA-54Na.

Dispersed Phase	Matrix Phase		
	TPS	EMAA	EMAA-54Na
TPS		0.64	0.35
EMAA	1.5		
EMAA-54Na	2.82		

## Data Availability

Not applicable.
